# MET exon 14 skipping defines a unique molecular class of non-small cell lung cancer

**DOI:** 10.18632/oncotarget.9541

**Published:** 2016-05-21

**Authors:** Difan Zheng, Rui Wang, Ting Ye, Su Yu, Haichuan Hu, Xuxia Shen, Yuan Li, Hongbin Ji, Yihua Sun, Haiquan Chen

**Affiliations:** ^1^ Department of Thoracic Surgery, Fudan University Shanghai Cancer Center, Shanghai, China; ^2^ Department of Oncology, Shanghai Medical College, Fudan University, Shanghai, China; ^3^ Department of Pathology, Fudan University Shanghai Cancer Center, Shanghai, China; ^4^ Institutes of Biomedical Sciences, Fudan University, Shanghai, China; ^5^ Cancer Research Laboratory, Fudan University Shanghai Cancer Center, Shanghai, China; ^6^ Innovation Center for Cell Signaling Network, Institute of Biochemistry and Cell Biology, Shanghai Institutes for Biological Sciences, Chinese Academy of Science, Shanghai, China; ^7^ Massachusetts General Hospital Cancer Center, Boston, MA, USA

**Keywords:** MET, non-small cell lung cancer, surgery, targeted therapy

## Abstract

**Purpose:**

Recurrent *MET* exon 14 splicing has been revealed in lung cancers and is a promising therapeutic target. Because we have limited knowledge about the natural history of *MET* mutant tumors, the current study was aiming to determine the clinical and pathological characteristics in non-small cell lung cancers (NSCLC).

**Results:**

Twenty-three patients (1.3%) were positive for *MET* exon 14 skipping. Patients with *MET* exon 14 skipping displayed unique characteristics: female, non-smokers, earlier pathology stage and older age. *MET* exon 14 skipping indicated an early event as other drivers in lung cancer, while *MET* copy number gain was more likely a late event in lung cancer. Overall survival (OS) of patients harboring *MET* exon 14 skipping was longer than patients with *KRAS* mutation. Almost four-fifths of the lung tumors with *MET* exon 14 skipping had *EGFR* and/or *HER2* gene copy number gains. *EGFR* inhibitor showed moderate antitumor activity in treatment of a patient harboring *MET* exon 14 skipping.

**Patients and Methods:**

From October 2007 to June 2013, we screened 1770 patients with NSCLC and correlated *MET* status with clinical pathologic characteristics and mutations in *EGFR*, *KRAS*, *BRAF*, *HER2*, and *ALK*. Quantitative Real-Time PCR was used to detect *MET* gene copy number gain. Immunohistochemistry (IHC) was also performed to screen *MET* exon 14 skipping. Clinicopathological characteristics and survival information were analyzed.

**Conclusions:**

*MET* exon 14 skipping was detected in 1.3% (23/1770) of the Chinese patients with NSCLC. *MET* exon 14 skipping defined a new molecular subset of NSCLC with identifiable clinical characteristics. The therapeutic *EGFR* inhibitors might be an alternative treatment for patients with *MET* mutant NSCLC.

## INTRODUCTION

Lung cancer is the leading cause of cancer deaths worldwide. Non–small cell lung cancer (NSCLC) comprises over 80% of all lung tumors. With better understanding of the major genetic alterations and signaling pathways involved of lung cancer, it has been classified into various subsets with different molecular and clinicopathologic characteristics [1]. Target-based therapeutics designed to specific molecular clusters have revolutionized lung cancer treatment. The most representative examples are gefitinib/erlotinib and crizotinib for lung adenocarcinomas harboring *EGFR* mutations and *ALK*/*ROS1* fusion, respectively [2, 3]. Even with broad genotyping, there remains 20%–40% of lung adenocarcinoma and 80% of lung squamous cell carcinoma without any known targetable oncogenic mutations [4–7].

*MET*, encoding the proto-oncogene tyrosine kinase *c-MET*, is the receptor for hepatocyte growth factor (HGF). The activation of this kinase by amplification and overexpression could promote cancer [8–10]. *MET* amplification is uncommon in NSCLCs and was observed in 2%–4% of previously untreated patients [8, 9, 11]. *MET* targeted therapies are currently undergoing early-phase clinical trial evaluations in lung cancer patients [12, 13].

Recently, *MET* gene exon 14 skipping was identified as a potential driver mutation in lung and colon tumors [14–18]. Strikingly, NSCLC patients harboring these genetic alterations have shown remarkable responses to *MET* inhibitor (crizotinib or cabozantinib) in several independent early-phase trials, suggesting that *MET* might be a novel druggable target in lung cancers [15–18]. The clinicopathologic characteristics of lung cancer patients with *MET* exon 14 skipping have not yet been described. These results provide us with an impetus to understand the natural history of *MET* mutant tumors.

Here, we demonstrated that *MET* exon 14 skipping is a novel oncogenic driver in lung cancers by clinical and pathological characteristics. This new molecular subset of patients had distinct features, allowing clinicians to select enriched subpopulations for genotyping and with whom randomized prospective clinical trials of targeted therapies could be efficiently performed.

## RESULTS

### Clinicopathological characteristics

From October 2007 to June 2013, we screened out 1770 qualified patients in this study cohort, including 1305 adenocarcinomas, 48 adenosquamous carcinomas and 417 squamous cell carcinomas. There were 991 (56.0%) male patients and 779 (44.0%) female patients. Ages below or above 60 years old were almost equally distributed. Seven hundred and seventy-nine patients were current smokers or former smokers; nine hundred and ninety-one patients were non-smokers. Obviously, most adenocarcinomas were female and non-smoking patients. Squamous cell carcinomas predominantly occurred in male and smoking patients. More detailed information was illustrated in Table 1.

**Table 1:** Clinical characteristics of 1770 patients with NSCLC

	Adenocarcinoma		Adenosquamous carcinoma		Squamous cell carcinoma	
	No.	%	No.	%	No.	%
**Total**	1305		48		417	
**Sex**						
Male	573	43.9%	34	70.8%	384	92.1%
Female	732	56.1%	14	29.2%	33	7.9%
**Age**						
> 60 y	657	50.3%	29	60.4%	228	54.7%
< 60 y	648	49.7%	19	39.6%	189	45.3%
**Smoking status**						
Smoker	401	30.7%	27	56.3%	351	84.2%
Never-smoker	904	69.3%	21	43.8%	66	15.8%
**Pathologic stage**						
**0**	32	2.5%	0	0.0%	0	0.0%
IA	510	39.1%	7	14.6%	70	16.8%
IB	171	13.1%	11	22.9%	104	24.9%
IIA	113	8.7%	3	6.3%	65	15.6%
IIB	43	3.3%	5	10.4%	47	11.3%
IIIA	352	27.0%	21	43.8%	125	30.0%
IIIB	30	2.3%	1	2.1%	4	1.0%
IV	54	4.1%	0	0.0%	2	0.5%
**Differentiation**						
Well	197	15.1%	0	0.0%	8	1.9%
Moderate	734	56.2%	16	33.3%	188	45.1%
Poor	374	28.7%	32	66.7%	221	53.0%
***EGFR* Mutation**						
Present	855	65.5%	20	41.7%	17	4.1%
Absent	450	34.5%	28	58.3%	400	95.9%
***KRAS* Mutation**						
Present	107	8.2%	6	12.5%	6	1.4%
Absent	1198	91.8%	42	87.5%	411	98.6%
***HER2* Insertion**						
Present	32	2.5%	1	2.1%	4	1.0%
Absent	1273	97.5%	47	97.9%	413	99.0%
***BRAF* Mutation**						
Present	20	1.5%	0	0.0%	0	0.0%
Absent	1285	98.5%	4	8.3%	417	100.0%
***ALK* Fusion**						
Present	70	5.4%	4	8.3%	2	0.5%
Absent	1235	94.6%	44	91.7%	415	99.5%
**RET Fusion**						
Present	18	1.4%	2	4.2%	0	0.0%
Absent	1287	98.6%	46	95.8%	417	100.0%
**ROS1 Fusion**						
Present	11	0.8%	0	0.0%	0	0.0%
Absent	1294	99.2%	48	100.0%	417	100.0%
**FGFR1/3 Fusion**						
Present	6	0.5%	0	0.0%	12	2.9%
Absent	1299	99.5%	48	100.0%	405	97.1%
***MET* Skipping**						
Present	21	1.6%	2	4.2%	0	0.0%
Absent	1284	98.4%	46	95.8%	417	100.0%
**PIK3CA Mutation**						
Present	22	1.7%	1	2.4%	12	7.5%
Absent	1270	98.3%	40	97.6%	147	92.5%
**CTNNB1 Mutation**						
Present	32	3.8%	1	2.4%	2	0.5%
Absent	815	96.2%	40	97.6%	371	99.5%

### Screening of *MET* exon 14 skipping in 1770 NSCLCs

**Figure 1:**
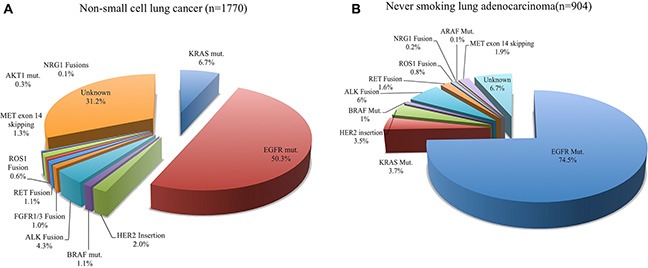
Mutational profiles in non-small cell lung cancer (**A**) Oncogenic driver mutations in 1770 non-small cell lung cancers; (**B**) Oncogenic driver mutations in 904 non-smoking lung adenocarcinomas.

The genetic alterations of 14 genes were screened in all NSCLCs (Table 1, Figure 1). Of 1770 patients, *MET* exon 14 skipping was detected in 23 patients (1.3%), which was the fourth most frequently identified driver in NSCLCs (Figure 1A) (Detailed data was shown in Supplementary Table S2). It was found in 21 (1.6%) of 1305 adenocarcinomas, and in two (4.2%) of 48 adenosquamous cell carcinomas. No mutant *MET* was detected in squamous carcinomas. We further revealed that 1.9% (17 of 904) of non-smoking lung adenocarcinomas (NSLAD) harbored *MET* exon 14 skipping (Figure 1B). Among 80 NSLADs that were negative for known oncogenic drivers, 21.25% of them harbored mutant *MET*.

### IHC analysis of *MET* exon 14 skipping positive NSCLCs

*MET* exon 14 skipping can only be detected using mRNA isolated from frozen tissues, which are commonly not available in clinical practice. Thus, we examined whether IHC could be utilized as a screening tool for detecting mutant *MET*. Of the 23 *MET* positive samples subjected to IHC, four scored as negative, nine as weak, eight as moderate, and two as strong. Another 64 pan-negative lung adenocarcinomas were also stained for *MET* protein; 42 scored as negative, 12 scored as weak, seven as moderate, and three as strong (Figure 3). The frequency of samples with *MET* positive in *MET* exon 14 skipping tumors was significantly higher than that in pan-negative samples (82.6% VS. 34.4%, Chi-Square test, *p <* 0.0001).

### *EGFR* and/or *HER2* copy number gains were common in *MET* Exon 14 skipping lung tumors

We showed that 18 of 23 (78.3%) lung tumors with *MET* exon 14 skipping had *EGFR* and/or *HER2* gene copy number gains. Among 19 invasive lung cancers, 94.7% of *MET* mutant tumors showed *EGFR* and/or *HER2* gene copy gains, but none of the cases with AAH/AIS harbored *EGFR* or *HER2* gene copy alterations (Table 3), indicating the pivotal role of *EGFR* or *HER2* in formation of invasive lung carcinoma in *MET* positive tumors. We also found that *EGFR* copy number gain was seen in all lung adenocarcinoma patients whose tumor harbored *MET* exon 14 skipping in TCGA (The Cancer Genome Atlas) cohort [6].

### Clinicopathologic characteristics of patients with *MET* exon 14 skipping

Here, we demonstrated that patients harboring *MET* exon 14 skipping defined a novel genetic subset of NSCLC. Patients with *MET* exon 14 skipping displayed unique characteristics: female, non-smokers, earlier pathology stage and older age (Table 2). Lepidic/acinar component were present in 58.8% of invasive lung adenocarcinomas with *MET* exon 14 skipping (Table 3). Atypical adenomatous hyperplasia (AAH)/ adenocarcinoma *in situ* (AIS) were observed in 17.4% (4 of 23) of *MET* positive tumors, indicating that it was an early event as other drivers in lung cancer. In contrast to patients with *MET* exon 14 skipping, patients with *MET* copy number gain were more prone to have late stage disease, poor differentiation, and acinar/solid/invasive mucinous adenocarcinoma (Table 4), indicating that *MET* copy number gain is a late event in lung cancer.

**Table 2:** Clinicopathologic characteristics of patients with *MET* exon 14 skipping in lung adenocarcinomas

	*MET* skipping		*EGFR*		*P*	*KRAS*		*P*	*ALK*		*P*	*Pan-negative		*P*
**Total**	21		855	%		107	%		70	%		165	%	
**Gender**														
Male	7	33.3	312	36.5		85	79.4		26	37.1		114	69.1	
Female	14	66.7	543	63.5	.823	22	20.6	.001	44	62.9	.802	51	30.9	.003
**Age**														
≥ 60 y	17	81.0	440	51.5		48	44.9		23	32.9		93	56.4	
< 60 y	4	19.0	415	48.5	.008	59	55.1	.003	47	67.1	.001	72	43.6	.035
**Smoking status**														
Smoking	4	19	182	21.3		73	68.2		16	22.9		102	61.8	
Never	17	81	673	78.7	.529	34	31.8	.001	54	77.1	.485	63	38.2	.001
**Tumor size**														
< 3 cm	13	61.9	560	65.5		52	48.6		38	54.3		75	45.5	
≥ 3 cm	8	38.1	289	33.8	.816	55	51.4	.341	32	45.7	.621	90	54.5	.171
**LN**														
0	15	71.4	553	64.7		68	63.6		31	44.3		91	55.2	
1	5	23.8	78	9.1		12	11.2		15	21.4		11	6.7	
2	1	4.8	219	25.6		26	24.3		19	27.1		60	36.4	
3	0	0	5	0.6	.037	1	0.9	.131	5	7.1	.056	3	1.8	.004
**Stage**														
**0**	4	19	16	1.9		0	0		1	1.4		5	3	
I	11	52.4	482	56.4		53	49.5		26	37.1		68	41.2	
II	5	23.8	88	10.3		21	19.6		12	17.1		25	15.2	
III	1	4.8	230	26.9		30	28.0		28	40.0		61	37.0	
IV	0	0	39	4.6	.001	3	2.8	.001	3	4.3	.001	6	3.6	.001
**Differentiation**													
Well	6	28.6	139	16.3		11	10.3		6	8.6		19	11.5	
Moderate	8	38.1	519	60.7		53	49.5		43	61.4		74	44.8	
Poor	7	33.3	197	23.0	.103	43	40.2	.077	21	30.	.038	72	43.6	.096
**Histology**														
AAH/AIS	4	19	43	5.0		1	0.9		1	1.4		9	5.5	
Lepidic	4	19	93	10.9		5	4.7		1	1.4		8	4.8	
Acinar	6	28.6	455	53.2		34	31.8		26	37.1		58	35.2	
Papillary	3	14.3	132	15.4		10	9.3		6	8.6		15	9.1	
Solid	3	14.3	85	9.9		33	30.8		20	28.6		58	35.2	
M-P	0	0.0	18	2.1		0	0		0	0		3	1.8	
IMA	1	4.8	14	1.6		22	20.6		16	22.9		10	6.1	
Enteric	0	0.0	0	0.0	.045	0	0	.001	0	0	.001	2	1.2	.028

Abbreviations: LN, lymph nodes; AAH, Atypical adenomatous hyperplasia; AIS, adenocarcinoma *in situ* (AIS); M-P, micropapillary; IMA, Invasive mucinous adenocarcinoma

**Table 3:** Characteristics of non-small cell lung cancer patients with *MET* exon 14 skipping

Cases	Sex	Age	Smoking	*P*.	T.	Subtype	Stage	*MET* IHC	*EGFR* CN	*HER2* CN	TTF1
1	F	67	Never	A	3.5	Acinar	IB	++	> 8	> 6	–
2	F	77	Never	A	2	Lepidic+Acinar	IB	+	> 4	2	+
3	M	66	Smoker	A	4	Papillary	IIA	++	> 4	2	+
4	F	74	Never	A	2.3	Lepidic	IA	++	> 6	2	+
5	F	54	Never	A	2	Acinar+Papillary	IB	++	> 10	2	–
6	M	60	Smoker	A	4.5	Solid	IB	++	> 7	2	+
7	F	75	Never	A	2.5	Solid	IB	+++	> 5	2	+
8	F	60	Never	A	4	Acinar+Lepidic	IIB	+	> 8	2	+
9	F	62	Never	A	3	Acinar+Solid	IA	++	> 5	2	+
10	F	49	Never	A	1	AAH	0	–	2	2	+
11	F	60	Never	A	2.8	Lepidic	IA	+	2	4	+
12	F	75	Never	A	2.5	IMA+Acinar	IA	+	> 5	2	–
13	M	76	Never	A	0.5	Acinar	IA	++	> 5	> 5	+
14	M	76	Smoker	A	2.8	Lepidic+Acinar	IA	–	> 5	3	+
15	M	67	Never	A	1.5	AIS	0	–	2	2	+
16	F	57	Never	A	0.8	AIS	0	+	2	2	+
17	F	73	Never	A	4	Papillary	IIA	+	2	2	+
18	F	47	Never	A	0.9	AIS	0	–	2	2	+
19	F	63	Never	A	2	Papillary+Micropapillary	IIA	+	> 7	2	+
20	M	68	Smoker	A	3.2	Solid	IIA	++	> 8	> 3	+
21	M	68	Never	A	3.5	Acinar+Solid	IIIA	+++	> 5	3	+
22	M	65	Smoker	AS	10	N.A.	IIB	+	> 4	2	+
23	F	77	Never	AS	1.5	N.A.	IA	+	> 5	> 4	+

Abbreviations: F, female, M, male; P, pathology; T. tumor size (cm); AAH, atypical adenomatous hyperplasia; AIS, adenocarcinoma *in situ*; IHC, *MET* immunohistochemistry; *EGFR* CN, *EGFR* gene copy by *EGFR*-FISH analysis; *HER2* CN, *HER2* gene copy by *HER2*-FISH analysis; N.A., not available; AAH, Atypical adenomatous hyperplasia; AIS, adenocarcinoma *in situ* (AIS).

**Table 4:** Comparison of clinicopathologic characteristics of patients with *MET* exon 14 skipping and with *MET* copy number gain in lung adenocarcinomas

	*MET* E14 skipping	%	*MET* copy number gain	%	*P*
**Total**	21		47		
**Gender**					
Male	7	33.33%	22	46.81%	
Female	14	66.67%	25	53.19%	0.427
**Age**					
≥ 60 y	17	80.95%	27	57.45%	
< 60 y	4	19.05%	20	42.55%	0.052
**Smoking status**					
Smoking	4	19.05%	14	29.79%	
Never-smoking	17	80.95%	33	70.21%	0.269
**Tumor size**					
< 3	13	61.90%	29	61.70%	
≥ 3	8	38.10%	18	38.30%	1.000
**Lymph Node status**					
0	15	71.43%	28	59.57%	
1	5	23.81%	5	10.64%	
2	1	4.76%	13	27.66%	
3	0	0.00%	1	2.13%	0.103
**Pathologic stage**					
**0**	4	19.05%	1	2.13%	
I	11	52.38%	23	48.94%	
II	5	23.81%	6	12.77%	
III	1	4.76%	16	34.04%	
IV	0	0.00%	1	2.13%	0.016
**Differentiation**					
Well	6	28.57%	3	6.38%	
Moderate	8	38.10%	25	53.19%	
Poor	7	33.33%	19	40.43%	0.043
**Predominant histology subtype**					
AAH/AIS	4	19.05%	1	2.13%	
Lepidic	4	19.05%	2	4.26%	
Acinar	6	28.57%	22	46.81%	
Papillary	3	14.29%	4	8.51%	
Solid	3	14.29%	11	23.40%	
Micropapillary	0	0.00%	1	2.13%	
IMA	1	4.76%	6	12.77%	0.046

Abbreviations: AAH, Atypical adenomatous hyperplasia; AIS, adenocarcinoma *in situ* (AIS); IMA, Invasive mucinous adenocarcinoma.

The proportion of precancerous or early lesions (AAH/AIS/Lepidic) (38.1%) in *MET* exon 14 skipping tumors was significantly higher than that of *EGFR* (15.9%), *KRAS* (5.6%), *HER2* (21.9%), *BRAF* (15%), *ALK* (2.9%), *RET* (5.6%), and pan-negative (10.3%) lung adenocarcinomas, indicating that mutant *MET* alone might be insufficient to transform normal cells but depend on other genetic alterations progressing into more aggressive carcinoma subtypes.

### Survival analyses

**Figure 2:**
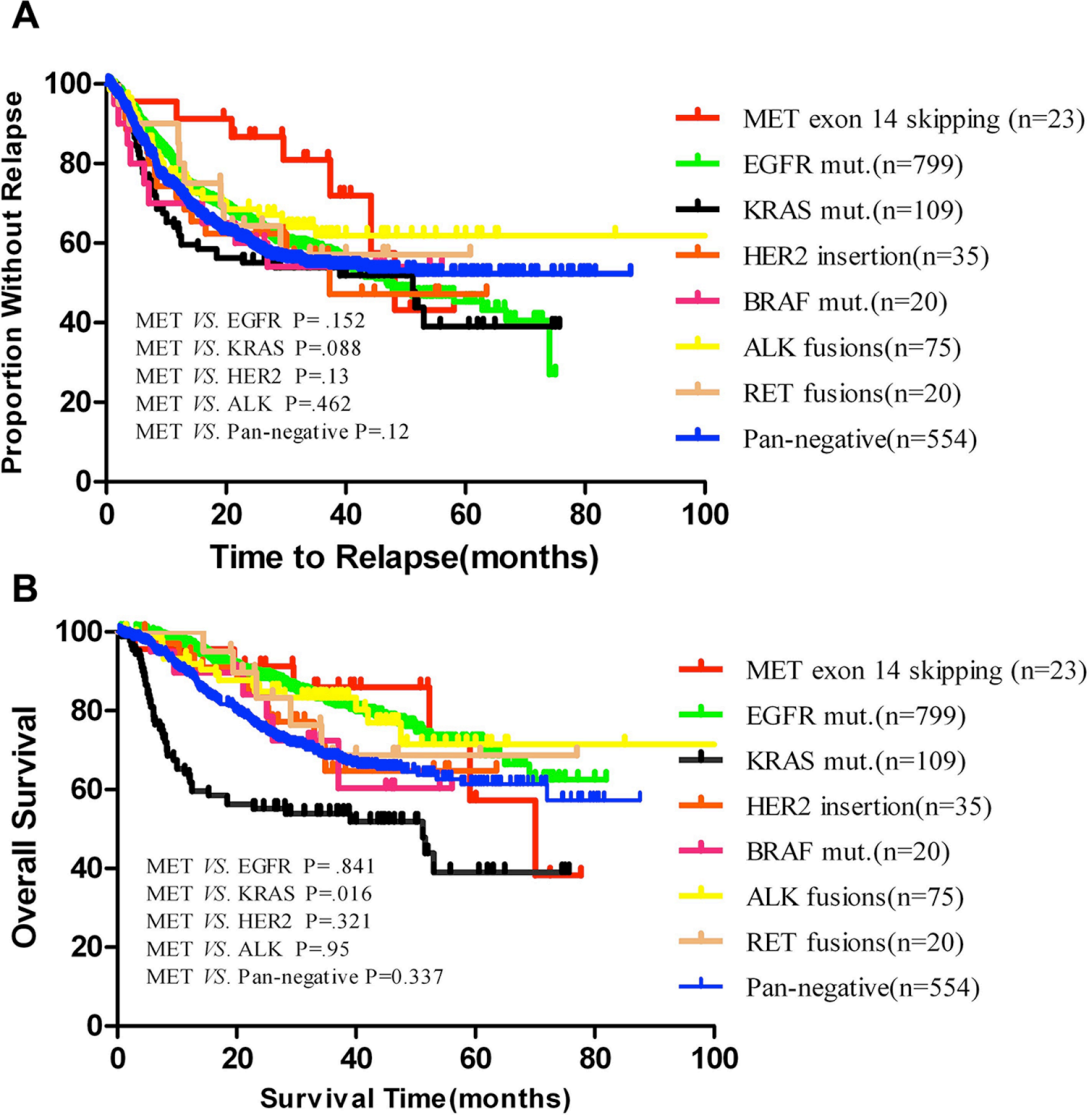
(**A**) Kaplan–Meier survival curves for relapse free according to *MET* exon 14 skipping or other known oncogenic drivers in 1635 NSCLC patients; (**B**) Overall survival according to *MET* exon 14 skipping or other known oncogenic drivers in 1393 NSCLC patients. Pan-negative indicates *EGFR*, *KRAS*, *HER2*, *BRAF*, *ALK*, *RET*, and *MET* negative.

The median relapse-free survival (RFS) was 46.2 months for *MET* compared with 48.2 months for *EGFR* (*P* = 0.152), 41.2 months for *KRAS* (*P* = 0.088), 39.4 months for *ALK* (*P* = 0.462), and 35 months for *HER2* (*P* = 0.13). The median overall survival (OS) was 70 months for *MET* compared with 67.2 months for *EGFR* (*P* = 0.841), 60 months for *KRAS* (*P* = 0.016), 52.7 months for *ALK* (*P* = 0.95), and 49.4 months for *HER2* (*P* = 0.321) (Figure 2). Multivariate analysis was not feasible because of the small number of deaths in the *MET* mutation group.

**Figure 3:**
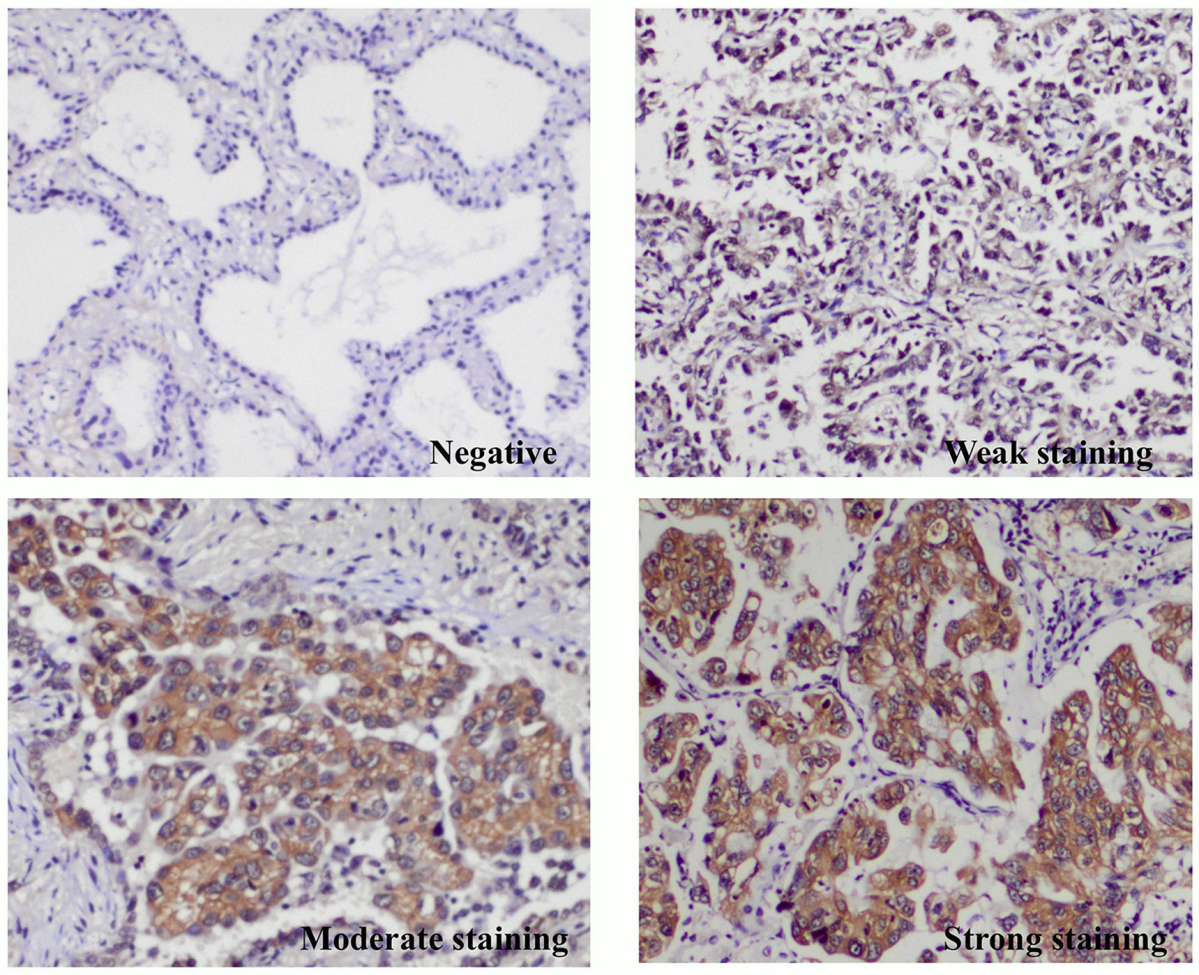
Representation of immunohistochemistry (IHC) staining of *MET* protein in pan-negative lung adenocarcinomas and *MET* exon 14 skipping positive non–small-cell lung cancers

### *EGFR* inhibitor demonstrates durable clinical response in a patient with advanced *MET* exon 14 skipping positive NSCLC

**Figure 4:**
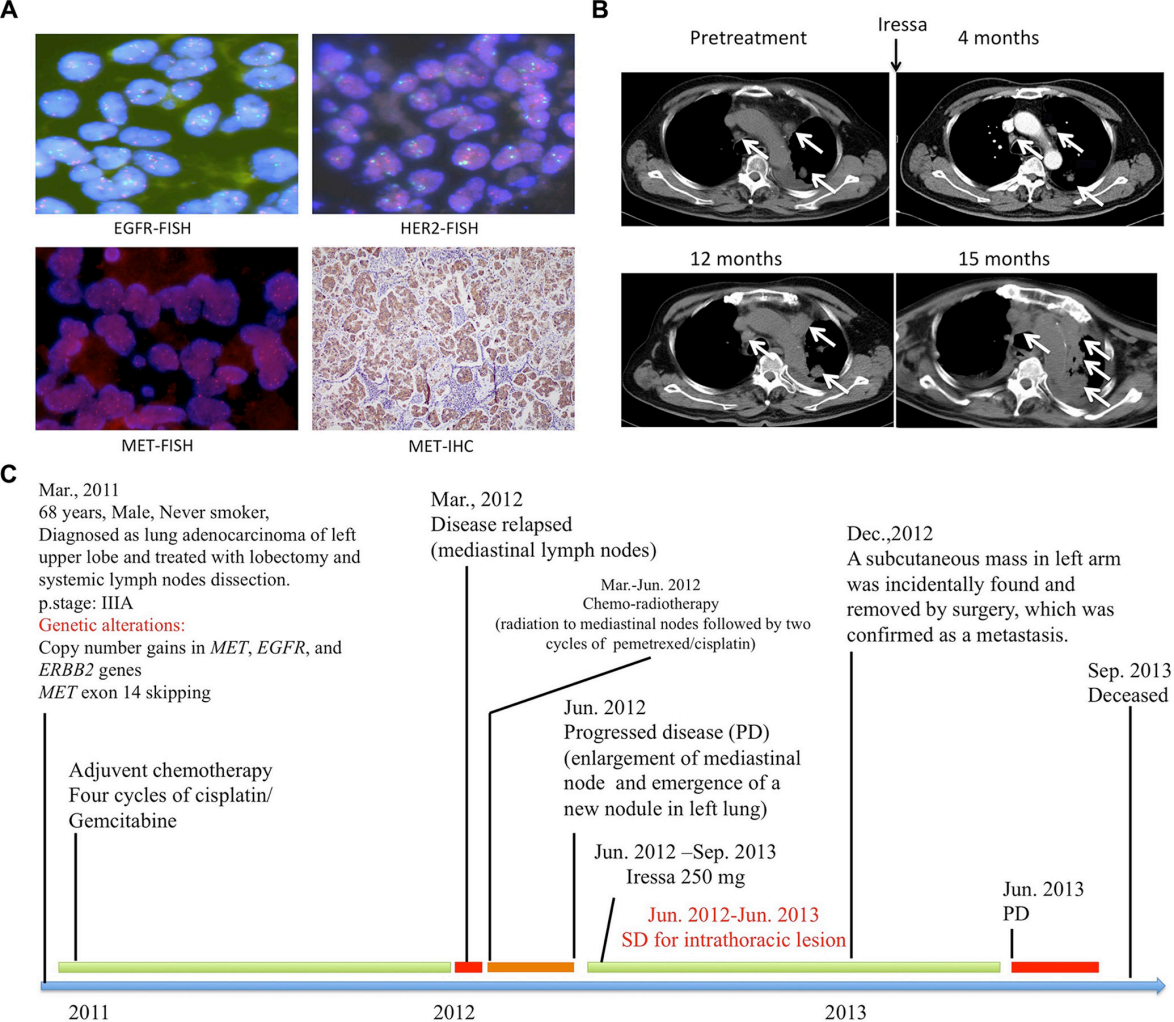
*EGFR* inhibitor shows durable clinical response in a patient harboring MET exon 14 skipping (**A**) FISH indicated that a *MET* exon 14 skipping positive non-smoker harbored *EGFR*, *HER2*, and *MET* copy number gains; IHC showed that this patient's tumor was positive for *MET* expression; (**B**) CT scan of the thorax demonstrating mediastinal lymph nodes enlargement and a left lobe mass before and after treatment with oral Iressa daily; (**C**) Timeline of the patient with *MET* exon 14 skipping lung.

Among the 23 NSCLC patients with *MET* exon 14 skipping in our cohort, a 68 year-old non-smoker with *MET*, *EGFR*, and *HER2* gene copy number gains in his tumor had relapsed. The patient showed a stable disease response to *EGFR* inhibitor Iressa, and the status had been maintained for 12 months (Figure 4). This modest clinical activity of Iressa might indicate that blockage of *EGFR* might serve as a salvage therapy for treatment of advanced *MET* exon 14 skipping positive patients if *MET* inhibitor is not available.

## DISCUSSION

In this study, we identified a subset of patients harboring *MET* exon 14 skipping as a novel oncogenic driver in lung cancers. As we previous reported [19], 90% of non-smoking lung adenocarcinomas (NSLAD) of East Asian populations were found to harbor known drivers that could be exploited as therapeutic targets. In this study, we examined 904 non-smoking NSLADs for all known oncogenic drivers. To the best of our knowledge, this study represents the most comprehensive analysis of mutation profiles in NSLADs. Of all non-smokers, 93.3% were found to carrying oncogenic driver mutations and 1.9% harbored *MET* exon 14 skipping. Given that *MET* exon 14 skipping defines a distinct subset of NSCLC patients characterized by female, non-smoker, and older age, further prospective clinical trials could exploit these clinical profiles to help clinicians select patients most likely to carry mutant *MET* and evaluate the clinical efficacy of *MET* inhibitor.

*MET* exon 14 skipping was not reckoned as a driver in lung cancer until the high-throughput sequencing revealed that this mutant kinase was mutually exclusive with known driver events [6, 7]. In TCGA's cohort, nine of 230 (3.9%) lung adenocarcinomas harbored *MET* exon 14 skipping mutation [6]. Seo JS, et al. reported three patients with *MET* exon 14 skipping mutation among a total of 200 patients, accounting for 1.5% in Asian populations [7]. Combined with our frequency of 1.6%, it seemed that Western populations had a slightly higher prevalence of the *MET* exon 14 skipping mutation than that of Asian populations. With the wide application of large-scale genome-sequencing technologies, many novel cancer-associated exon-skipping events have been identified [6, 7]. The copy number of known driver oncogenes, including *MET*, remains unchanged in most lung tumors [6, 7].

Garrett M.F. et al. demonstrated that NIH-3T3 cells stably expressed mutant *MET* showed anchorage-independent growth. This constitutive activity was sensitive to *MET* inhibitors in NIH-3T3 cells harboring *MET* exon 14 alterations [23]. It is interesting that five independent studies almost simultaneously reported the efficacy of *MET* inhibitors (crizotinib and capmatinib) for treating patients harboring *MET* exon 14 skipping [15–18, 23]. Of the five studies, 11 patients are evaluable for responses to the *MET* inhibitor. The response rate was 83.3% (nine of 12 lesions) and the median progression free survival was five months (range of two to 13 months). The antitumor activity shown in early phase clinical trials strongly supports *MET* exon 14 skipping as a bona fide target of the *MET* inhibitor. However, the responses to the *MET* inhibitor are quite heterogeneous. There still remains a substantial amount of tumor cells that are intrinsically resistant to the *MET* tyrosine kinase inhibitor. The molecular basis of the response heterogeneity is largely unknown, and no biomarker is available for predicting a response.

Remarkably, almost four-fifths of the patients with *MET* exon 14 skipping in our cohort had *EGFR* and/or *HER2* gene copy number gains. Whether this observed consequence was a coincidence or an intrinsic connection remained unclear. One patient harboring *MET* exon 14 skipping simultaneously with *EGFR*, *HER2* gene copy number gains received Iressa and maintained his disease for 12 months. It might be difficult to Figure out which alternation of genes played a more important role. Specific *MET* inhibitors should be an excellent choice according to the reported data; *EGFR*-TKIs, however may come to rescue as a replacement, especially when patients have had *EGFR* gene alternations. Since these three genes are essential in cell signaling, affecting various kinds of activities in cells such as survival, proliferation and invasion, completely understanding of the interaction of these genes is a necessity, and further experiments are urgently demanded. Future possible therapeutic strategy may hide in this network by double or triple targeting these molecules.

In summary, *MET* exon 14 skipping defined a new molecular subset of NSCLC with identifiable clinical characteristics. These patients are typically older, female, and non-smokers who might benefit from *MET* targeted therapy. Most of the lung tumors with *MET* exon 14 skipping had *EGFR* and/or *HER2* gene copy number gains. Additionally, the therapeutic *EGFR* inhibitors might be an alternative treatment for patients with *MET* mutant NSCLC.

## MATERIALS AND METHODS

### Patients and tissues

From October 2007 to June 2013, a total of 1770 frozen surgically resected NSCLC tumor tissues, including 1305 lung adenocarcinomas, 48 adenosquamous carcinoma and 417 squamous cell carcinomas, were prospectively collected in the Department of Thoracic Surgery of Fudan University Shanghai Cancer Center. This study was approved by the Institutional Review Board of the Fudan University Shanghai Cancer Center. All patients provided written informed consent. Two pathologists (Y.L. and X.X.S.) confirmed the diagnosis by H&E staining and verified by immunohistochemistry as *TTF1*, *P63*, *Nepsin A* and *CK5/6*. DNA and RNA were collected as previously reported [19, 20].

### Mutational analyses

As previously reported [5, 19–21], mutational status of *EGFR*, *KRAS*, *HER2*, *BRAF*, *CTNNB1*, *PIK3CA*, *ALK*, *RET*, *ROS1*, *NRG1*, *FGFR1*, *FGFR2*, and *FGFR3* was detected. *MET* (exon 13 to 21) was PCR amplified using cDNA for direct sequencing and exon 14 deleted cases were verified by sequencing of the PCR product of *MET* exon 13 to 15. Primers were shown in Supplementary Table S1. Quantitative Real-Time PCR was used to detect *MET* gene copy number gain as previously reported [22].

### Immunohistochemistry (IHC)

Standard 5-μm formalin-fixed paraffin-embedded sections were subjected to IHC analysis using anti-*MET* (Cell Signaling Technology), *P40* (ΔP63, Fuzhou Maxim Biotech, Ltd. China), and *TTF-1* (clone: SPT24, Ready- to-use antibody, Fuzhou Maxim Biotech, Ltd. China) antibodies.

### Clinicopathological characteristics

Clinical and pathologic data were prospectively collected for analyses, including age at diagnosis, sex, smoking history, histologic type, pathologic TNM stage, tumor size, and tumor differentiation. Patients were observed in clinic or by telephone for disease recurrence and survival from the date of diagnosis. Of the 1770 NSCLC patients, 56 patients who were incidentally found to have pleural metastasis were excluded from survival analysis. Seventy-nine patients of loss to follow-up were also excluded. A total of 1635 NSCLC patients were included for analysis with a median follow-up time of 32.83 months.

### Statistical analysis

Associations between mutations and clinical and pathologic characteristics were analyzed by the χ2 test or Fisher's exact test. Survival curves were estimated using the Kaplan-Meier method, with differences in survival assessed by the log-rank test. Data were analyzed using Statistical Package for the Social Sciences Version 19.0 Software (SPSS Inc.). The two-sided significance level was set at *p <* 0.05.

## SUPPLEMENTARY FIGURES AND TABLES


