# Supplementary material for: MET exon 14 skipping defines a unique molecular class of non-small cell lung cancer
Source: Oncotarget. 2016 May 21;7(27):41691–702. doi: 10.18632/oncotarget.9541 (PMC5173088; doi:10.18632/oncotarget.9541)
Supplement: Supplementary file 1 [file oncotarget-07-41691-s001.pdf]

MET exon 14 skipping defines a unique molecular class of non-small cell lung cancer

Supplementary Materials

Supplementary Table S1: Primers used in this study

Primers to amplify targeted genomic region for surveyor assay		
Target	Forward primer (5′ >> 3′)	Reverse primer (5′ >> 3′)
met-genome-human-DNA	TCTATCATGGCTAAATGCTGACT	TGTAACTTAACTCTTCCTGGATTG
**Primers for detecting MET copy number variation**		
MET-DNA	CCATCCAGTGTCTCCAGAAGTG	TTCCCAGTGATAACCAGTGTGTAG
MTHFR	CCATCTTCCTGCTGCTGTAACTG	GCCTTCTCTGCCAACTGTCC

Supplementary Table S2: PCR results of patients with *MET* exon 14 skipping

Cases	PCR	Sequence
1	c.2890_3031del	…GTGGCTGAAAAAGAGAAAGCAAATTAAAGAT //exon 14// AGTTTCCTAATTCATCTCAGAACGGTTCATG…
2	c.2889_3031del	…TGTGGCTGAAAAAGAGAAAGCAAATTAAAGA //exon 14// AGTTTCCTAATTCATCTCAGAACGGTTCATG…
3	c.2890_3031del	…GTGGCTGAAAAAGAGAAAGCAAATTAAAGAT //exon 14// AGTTTCCTAATTCATCTCAGAACGGTTCATG…
4	c.2889_3031del	…TGTGGCTGAAAAAGAGAAAGCAAATTAAAGA //exon 14// AGTTTCCTAATTCATCTCAGAACGGTTCATG…
5	c.2890_3031del	…GTGGCTGAAAAAGAGAAAGCAAATTAAAGAT //exon 14// AGTTTCCTAATTCATCTCAGAACGGTTCATG…
6	c.2889_3031del	…TGTGGCTGAAAAAGAGAAAGCAAATTAAAGA //exon 14// AGTTTCCTAATTCATCTCAGAACGGTTCATG…
7	c.2890_3031del	…GTGGCTGAAAAAGAGAAAGCAAATTAAAGAT //exon 14// AGTTTCCTAATTCATCTCAGAACGGTTCATG…
8	c.2890_3031del	…GTGGCTGAAAAAGAGAAAGCAAATTAAAGAT //exon 14// AGTTTCCTAATTCATCTCAGAACGGTTCATG…
9	c.2890_3031del	…GTGGCTGAAAAAGAGAAAGCAAATTAAAGAT //exon 14// AGTTTCCTAATTCATCTCAGAACGGTTCATG…
10	c.2889_3031del	…TGTGGCTGAAAAAGAGAAAGCAAATTAAAGA //exon 14// AGTTTCCTAATTCATCTCAGAACGGTTCATG…
11	c.2890_3031del	…GTGGCTGAAAAAGAGAAAGCAAATTAAAGAT //exon 14// AGTTTCCTAATTCATCTCAGAACGGTTCATG…
12	c.2890_3031del	…GTGGCTGAAAAAGAGAAAGCAAATTAAAGAT //exon 14// AGTTTCCTAATTCATCTCAGAACGGTTCATG…
13	c.2890_3031del	…GTGGCTGAAAAAGAGAAAGCAAATTAAAGAT //exon 14// AGTTTCCTAATTCATCTCAGAACGGTTCATG…
14	c.2890_3031del	…GTGGCTGAAAAAGAGAAAGCAAATTAAAGAT //exon 14// AGTTTCCTAATTCATCTCAGAACGGTTCATG…
15	c.2890_3031del	…GTGGCTGAAAAAGAGAAAGCAAATTAAAGAT //exon 14// AGTTTCCTAATTCATCTCAGAACGGTTCATG…
16	c.2889_3031del	…TGTGGCTGAAAAAGAGAAAGCAAATTAAAGA //exon 14// AGTTTCCTAATTCATCTCAGAACGGTTCATG…
17	c.2889_3031del	…TGTGGCTGAAAAAGAGAAAGCAAATTAAAGA //exon 14// AGTTTCCTAATTCATCTCAGAACGGTTCATG…
18	c.2889_3031del	…TGTGGCTGAAAAAGAGAAAGCAAATTAAAGA //exon 14// AGTTTCCTAATTCATCTCAGAACGGTTCATG…
19	c.2890_3031del	…GTGGCTGAAAAAGAGAAAGCAAATTAAAGAT //exon 14// AGTTTCCTAATTCATCTCAGAACGGTTCATG…
20	c.2890_3031del	…GTGGCTGAAAAAGAGAAAGCAAATTAAAGAT //exon 14// AGTTTCCTAATTCATCTCAGAACGGTTCATG…
21	c.2890_3031del	…GTGGCTGAAAAAGAGAAAGCAAATTAAAGAT //exon 14// AGTTTCCTAATTCATCTCAGAACGGTTCATG…
22	c.2890_3031del	…GTGGCTGAAAAAGAGAAAGCAAATTAAAGAT //exon 14// AGTTTCCTAATTCATCTCAGAACGGTTCATG…
23	c.2890_3031del	…GTGGCTGAAAAAGAGAAAGCAAATTAAAGAT //exon 14// AGTTTCCTAATTCATCTCAGAACGGTTCATG…
